# Clinical risk factors for portal hypertension-related complications in systemic therapy for hepatocellular carcinoma

**DOI:** 10.1007/s00535-024-02097-9

**Published:** 2024-04-07

**Authors:** Kisako Fujiwara, Takayuki Kondo, Kentaro Fujimoto, Sae Yumita, Keita Ogawa, Takamasa Ishino, Miyuki Nakagawa, Terunao Iwanaga, Satoshi Tsuchiya, Keisuke Koroki, Hiroaki Kanzaki, Masanori Inoue, Kazufumi Kobayashi, Soichiro Kiyono, Masato Nakamura, Naoya Kanogawa, Sadahisa Ogasawara, Shingo Nakamoto, Tetsuhiro Chiba, Jun Koizumi, Jun Kato, Naoya Kato

**Affiliations:** 1 Department of Gastroenterology, Graduate School of Medicine, Chiba University, 1-8-1, Inohana, Chuo-ku, Chiba, 260-8670 Japan; 2 Ultrasound Center, Chiba University Hospital, Chiba, Japan; 3 Department of Radiology, Graduate School of Medicine, Chiba University, Chiba, Japan

**Keywords:** Systemic therapy, Hepatocellular carcinoma, Contrast-enhanced computed tomography, Portal hypertension, Decompensation

## Abstract

**Background:**

During systemic therapy, the management of portal hypertension (PH)-related complications is vital. This study aimed to clarify factors associated with the incidence and exacerbation of PH-related complications, including the usefulness of contrast-enhanced computed tomography (CECT) in the management of PH-related complications during systemic therapy.

**Methods:**

A total of 669 patients who received systemic therapy as first-line treatment (443 patients for sorafenib, 131 for lenvatinib, and 90 for atezolizumab/bevacizumab [ATZ/BEV]) were enrolled in this retrospective study. Additionally, the lower esophageal intramural vessel diameters (EIV) on CECT and endoscopic findings in 358 patients were compared.

**Results:**

The cutoff values of the EIV diameter on CECT were 3.1 mm for small, 5.1 mm for medium, and 7.6 mm for large varices, demonstrating high concordance with the endoscopic findings. esophageal varices (EV) bleeding predictors include EIV ≥ 3.1 mm and portal vein tumor thrombosis (PVTT). In patients without EV before systemic therapy, factors associated with EV exacerbation after 3 months were EIV ≥ 1.9 mm and ATZ/BEV use. Predictors of hepatic encephalopathy (HE) include the ammonia level or portosystemic shunt diameter ≥ 6.8 mm. The incidence of HE within 2 weeks was significantly higher (18%) in patients with an ammonia level ≥ 73 μmol/L and a portosystemic shunt ≥ 6.8 mm. The exacerbating factors for ascites after 3 months were PVTT and low albumin levels.

**Conclusions:**

Careful management is warranted for patients with risk factors for exacerbation of PH-related complications; moreover, the effective use of CECT is clinically important.

**Supplementary Information:**

The online version contains supplementary material available at 10.1007/s00535-024-02097-9.

## Introduction

Hepatocellular carcinoma (HCC) remains an important cause of cancer-related mortality, and in recent years, its incidence has continued to incline [1]. Most patients with HCC are diagnosed with stage B or C in the Barcelona Clinic Liver Cancer staging system, with a reported 5-year survival rate of 16% [2]. As an alternative to multi-kinase inhibitors such as sorafenib (SOR) or lenvatinib (LEN) for the treatment of HCC, the combination of atezolizumab, a fully humanized anti-programmed death-ligand 1 (PD-L1), and bevacizumab, a vascular endothelial growth factor (VEGF) targeting antibody (ATZ/BEV), was recently approved as first-line therapy, with a median overall survival benefit of 17 months [3].

The impact of the use of VEGF and immune checkpoint inhibitors on portal hypertension (PH) has been elucidated in various studies [4–9]. However, limited data exist regarding the impact of systemic therapy on PH in real-world practice [3].

The management of PH-related complications, such as esophageal variceal hemorrhage, hepatic encephalopathy (HE), and ascites during HCC treatment can lead to treatment discontinuation and affect patient prognosis [10, 11]. The presence of a portosystemic shunt contributes to the development of HE and is a predictor of poor prognosis [12]. In patients with HCC, a sufficient evaluation of PH remains a major problem, and periodic endoscopy is preferred for screening esophageal varices (EV), especially in patients with vascular invasion [8]. However, little is known regarding the effective management of PH-related complications without interfering with HCC treatment. Contrast-enhanced computed tomography (CECT) is often used to assess disease status in patients with HCC undergoing systemic therapy. CECT has been reported to be useful for screening EV [13]. Additionally, other studies have reported the efficacy of CECT in evaluating portosystemic shunts [14]. We previously reported the usefulness of CECT in the management of EV in patients with HCC [15]. However, further validation is required in patients with HCC undergoing systemic therapy. Therefore, this study aimed to identify factors that cause or exacerbate PH-related complications and verify the usefulness of CECT in the management of PH-related complications in patients with HCC receiving systemic therapy.

## Materials and methods

### Study design

This retrospective study enrolled consecutive patients with HCC who received systemic therapy as a first-line treatment between 2009 and 2022, and clinical findings were obtained from our institutional database. The exclusion criteria for this study were as follows: patients who did not receive CECT within 3 months before systemic therapy and those with advanced cancer other than HCC.

This study was conducted in accordance with the principles of the Declaration of Helsinki and was approved by the Ethics Committee of the Graduate School of Medicine, Chiba University. The requirement for written informed consent was waived due to the retrospective nature of the study, and informed consent was obtained in the form of an opt-out on the website.

The endpoints were the incidence of PH-related complications, such as EV bleeding, HE, ascites, and exacerbation of EV during the observation period. The observation period for EV bleeding was from the initiation of the first-line systemic therapy to the date of EV bleeding, the start of second-line systemic therapy, prophylactic treatment for EV, last hospital visit, or death. The observation period for HE was within 2 weeks after the initiation of the first-line systemic therapy, and that for the exacerbation of EV and ascites was between the start of and 3 months after the first-line systemic therapy. Furthermore, a comparison was performed between the CECT and endoscopic findings in patients who underwent endoscopy within 3 months of CECT.

### CECT and assessment of EV and portosystemic shunt

CECT was performed using a 64-detector CT scanner (Aquilion 64, Toshiba), an 80-detector CT scanner (Aquilion Prime), and a 320-detector CT scanner (Aquilion ONE, Toshiba). The contrast agent was mechanically injected through a peripheral vein at a 100-mL dose and a 3 mL/s injection. Imaging was performed in three phases: the hepatic artery, portal vein, and equilibrium phases. EVs were defined as intramural enhancing nodular tubular structures. For EV, the diameter of the contrasted vessel protruding into the esophageal mucosa was measured perpendicularly to the mucosal surface using a 5-mm slice thickness axial CECT in the portal vein phase, and the maximum short-axis diameter of the lower esophageal intramural vessel (EIV) was recorded. The natural connection between the portal circulation and the systemic venous system was defined as the portosystemic shunt, excluding the EV, and the maximum diameter was recorded. To assess interobserver variability, all CT scans were reviewed by two hepatologists. One physician was a supervisor with more than 15 years of clinical experience, and the other was a specialist with 8 years of clinical experience.

### Definitions

A diagnosis of EV bleeding was established when the nature of the gastrointestinal bleeding, such as hematemesis, hematochezia, or melena, was confirmed to be of variceal origin via endoscopy. On upper gastrointestinal endoscopy, the EV severity was classified into the following four groups based on a previous report [16]: (i) absence of varices, (ii) small varices (F1), (iii) medium varices (F2), and (iv) large varices (F3). In this study, portal vein tumor thrombosis (PVTT) is defined as a macrovascular invasion (VP2, tumor thrombus in a second branch of the portal vein; VP3, tumor thrombus in the first branch of the portal vein; and VP4, tumor thrombus in the trunk of the portal vein). A history of EV treatment was defined as a patient who had undergone endoscopic injection sclerotherapy or variceal ligation for EV prior to systemic therapy.

The West Haven grading system was utilized to evaluate HE, and a grade II, or higher was treated as HE. The portosystemic shunt included the left gastric vein, posterior gastric vein, short gastric vein, spleno-renal shunt, and gastro-renal shunt excluding the EV. Incidence of ascites was defined as the accumulation of ascites on CECT or abdominal paracentesis for the first time after systemic therapy. Cirrhosis was diagnosed through a combination of clinical symptoms and findings on clinical examination, radiographic imaging, or liver biopsy. HCC with a liver occupancy of 50% or more was defined as a high total tumor volume HCC [17]. To determine the treatment response 3 months after systemic therapy, the Response Evaluation Criteria in Solid Tumors version 1.1 was used.

### Statistical analysis

All data were expressed as mean ± standard deviation or percentage. The Student’s *t* test, Mann–Whitney *U* test, or paired *t* test, and one-way analysis of variance, as appropriate, were used to analyze the continuous variables. Categorical variables were analyzed using the Chi-square test. Using the Kaplan–Meier method, the cumulative overall survival and variceal bleeding rates were calculated, and risk factors for EV bleeding were assessed using Cox regression analysis. Multivariate analysis for the exacerbation of EV, HE, and ascites was performed using logistic regression analysis. In multivariate analysis, variables were included if the *p* value was *p* < 0.01 in univariate analysis, and Child–Pugh score and ALBI score were not included because of the potential confounding factors. Optimal cutoff values were calculated using the area under the receiver operating characteristic curve (AUC) analysis, and statistical significance was set at *p* < 0.05. However, Bonferroni correction was used for multiple comparisons (categorical variables in the groups), and statistical significance was set at *p* < 0.01. The SAS version 9.4 (SAS Institute, Cary, NC, USA) was used for the statistical data analysis. Classification and regression tree (CART) analysis was performed using the R-powered data tool exploratory.

## Results

### Patient characteristics

**Fig. 1:**
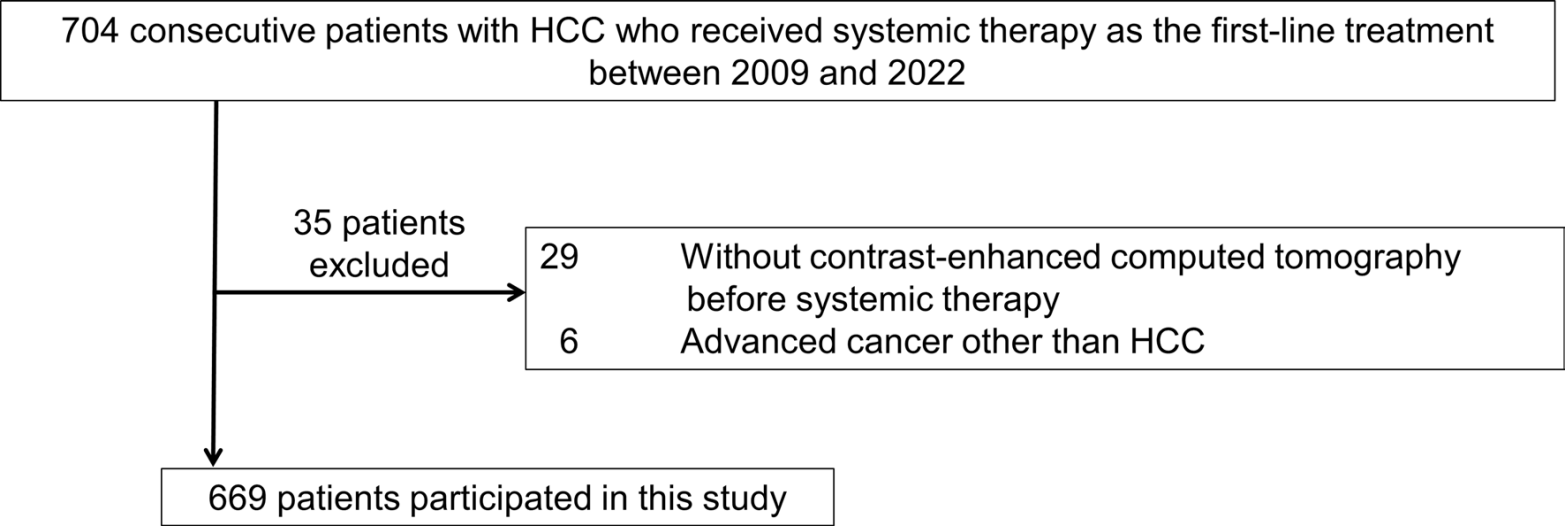
Flow diagram of the study participants. *HCC* hepatocellular carcinoma

A total of 669 patients were included in this study (448 with SOR, 131 with LEN, and 90 with ATZ/BEV). The study flowchart is shown in Fig. 1. The median observation duration was 0.7 months. The patient characteristics for each type of systemic therapy are shown in Table 1. Viral hepatitis was significantly lower in the ATZ/BEV group than in the SOR group; however, other background factors were generally balanced.

**Table 1:** Patient characteristics

	SOR	LEN	ATZ/BEV	*p* value
Number of patients	448	131	90	
Age (≥ 75 years)	167 (37.3%)	50 (36.2%)	43 (47.8%)	0.18
Etiology virus	280 (62.5%)	69 (52.7%)	30 (33.3%)	< 0.01
Sex (female)	357 (20.3%)	107 (18.3%)	13 (14.4%)	0.42
Liver cirrhosis	292 (65.2%)	92 (70.2%)	57 (63.3%)	0.48
PVTT	112 (25.0%)	43 (32.8%)	21 (23.3%)	0.16
EHM	151 (33.7%)	34 (26.0%)	24 (26.7%)	0.15
Child–Pugh A/B	366/81	109/22	82/8	0.09
ALBI score	− 2.28 ∓ 0.49	− 2.21 ∓ 0.52	− 2.40 ∓ 0.44	0.01
MELD score	4.8 ∓ 3.9	5.0 ∓ 3.8	4.6 ∓ 2.8	0.72
Ascites	67 (15.0%)	18 (13.7%)	12 (13.3%)	0.16
History of treatment for EV	25 (5.6%)	12 (9.5%)	8 (9.2%)	0.21

*ALBI* Albumin-Bilirubin, *ATZ/BEV* atezolizmab/bevacizumab, *EHM* extrahepatic metastasis, *EV* esophageal varices, *LEN* Lenvatinib, *MELD* Model for End-Stage Liver Disease, *PVTT* portal vein tumor thrombosis, SOR Sorafenib

Of the 669 consecutive patients, 358 underwent endoscopy within 6 months of CECT. The EIV diameters based on the classification of endoscopic varices were as follows: no varices: 0.8 ± 1.2 mm; small varices (F1): 3.5 ± 1.2 mm; medium varices (F2): 7.4 ± 2.0 mm; and large varices (F3): 7.9 ± 3.0 mm. The most optimal cutoff values were F1: 3.0 mm (AUC = 0.950); F2: 5.1 mm (AUC = 0.992); and F3: 7.6 mm (AUC = 0.976).

### EV bleeding after systemic therapy

During first-line treatment, a total of 41 patients manifested EV bleeding, and eight received prophylactic treatment for EV during the follow-up period. The cumulative EV bleeding rates were 1.9% at 3 months and 4.1% at 6 months. In patients with EV prior to systemic therapy, the cumulative bleeding rate (6.0% at 3 months and 11.6% at 6 months) was significantly higher than in those without EV (0.2% at 3 months and 1.1% at 6 months, *p* < 0.01). Table 2 shows the predictive factors for EV bleeding according to the univariate analysis. Multivariate analysis revealed that significant predictors of EV bleeding were an EIV diameter ≥ 3.1 mm (*p* < 0.01) and PVTT (*p* < 0.01) (Table 2). The EIV diameter was also a significant factor in analyses restricted to Child–Pugh score 5 patients (Supplementary Table 1).

**Table 2:** Cox regression analyses of predictive factors for variceal bleeding

	Univariate hazard ratio (95% confidence interval)	*P* value	Multivariate hazard ratio (95% confidence interval)	*P *value
Age (≥ 75 years)	0.49 (0.24–0.99)	0.05	–	
Female sex	0.67 (0.26–1.72)	0.41	–	
Etiology virus	1.47 (0.76–2.85)	0.25	–	
Liver cirrhosis	4.21 (1.65–10.75)	< 0.01	–	
PVTT	3.84 (2.05–7.19)	< 0.01	4.01 (2.02–7.99)	< 0.01
EHM	0.58 (0.27–1.26)	0.17	–	
LEN	0.98 (0.41–2.36)	0.96	–	
ATZ/BV	0.42 (0.10–1.77)	0.24	–	
High total tumor volume	1.34 (0.32–5.59)	0.69	–	
Ascites	3.71 (1.73–7.97)	< 0.01	–	
History of treatment for HCC	0.70 (0.31–1.59)	0.39	–	
History of treatment for EV	2.79 (1.17–6.67)	0.02	–	
PPI	1.40 (0.70–2.82)	0.34	–	
Findings on contrast enhanced CT				
Diameter of intramural vessel in esophagus ≥ 3.1(mm)	14.81 (6.55–33.49)	< 0.01	10.96 (4.21–28.56)	< 0.01
Diameter of portosystemic shunt ≥ 3.0(mm)	1.95 (1.05–3.61)	0.03	–	
Laboratory data				
Alanine aminotransferases (U/L)	1.01 (1.00–1.02)	0.11	–	
Bilirubin (mg/dL)	2.06 (1.37–3.11)	< 0.01	–	
Prothrombin time (international normalized ratio)	1.55 (0.48–5.05)	0.47	–	
Albumin (g/dL)	0.31 (0.16–0.58)	< 0.01	–	
Platelets (10^9^/L)	0.91 (0.86–0.97)	< 0.01	–	
Ammonia (μg/dL)	1.01 (1.00–1.02)	< 0.01	–	
Alfa fetoprotein (ng/mL)	1.00 (1.00–1.00)	0.07	–	
ALBI score	3.84 (1.87–7.89)	< 0.01	–	
Child-Pugh B	2.72 (1.32–5.61)	< 0.01	–	

*ALBI* Albumin-Bilirubin, *ATZ/BEV* atezolizmab/bevacizumab, *CT* computed tomography, *EHM* extrahepatic metastasis, *EV* esophageal varices, *HCC* hepatocellular carcinoma, *LEN* Lenvatinib, *NSAIDs* Non-Steroidal Anti-Inflammatory Drugs, Portosystemic shunt maximum diameter of portosystemic shunt other than esophageal varices, *PPI* Proton pump inhibitor, *PVTT* portal vein tumor thrombosis

The cumulative overall survival in patients with variceal bleeding (57.1% at 6 months and 38.6% at 1 year) was significantly lower than in those patients without EV bleeding (72.4% at 6 months and 51.4% at 1 year; *p* < 0.01).

Considering the treatment response for HCC, progressive disease (PD) after 3 months was not associated with EV bleeding during systemic therapy (*p* = 0.38). Classifying PVTT into VP2-3 and VP4, there was no significant difference in EV bleeding between the group with VP2-3 (5.8% at 3 months and 12.2% at 6 months) and VP4 (6.2% at 3 months and 9.2% at 6 months, *p* = 0.82).

### Exacerbation of EV 3 months after systemic therapy

Of the 669 patients, a total of 439 enrolled patients underwent CECT at 3 months after systemic therapy. In these 439 patients, the EIV diameter was significantly dilated 3 months after systemic therapy compared to that before (before vs. after 3 months; 1.4 mm [0–3.0] vs. 1.7 mm [0–3.4], *p* < 0.01). Using the CECT classification, the exacerbation rates of EV 3 months after systemic therapy were as follows: 14.1% (46/326) without EV before systemic therapy, 24.7% (19/77) with F1, and 13.6% (3/20) with F2.

To clarify the predictors of EV exacerbation in patients without EV, we analyzed the data of 326 patients without EV based on CECT prior to systemic therapy (Table 3). Significant predictors of EV exacerbation in patients without EV were identified in the multivariate analysis, with odds ratios of 8.06 (95% confidence interval [CI] 3.91–16.64, *p* < 0.01) for ATZ/BEV use and 4.06 (95% CI 1.88–8.78, *p* < 0.01) for an EIV diameter ≥ 1.9 mm. ATZ/BEV use was also a significant factor in analyses restricted to Child–Pugh score 5 patients (Supplementary Table 2).

**Table 3:** Predictors for EV exacerbation rate after 3 months (univariate analysis)

	Without EV exacerbation after 3 months	EV exacerbation after 3 months	*P* value
Number of patients	280	46	
Age (≥ 75 years)	118 (42.1%)	25 (54.4%)	0.12
Female sex	53 (18.9%)	4 (8.7%)	0.09
Etiology Virus	150 (53.6%)	24 (52.2)	0.86
Liver cirrhosis	150 (53.6%)	32 (69.6%)	0.04
PVTT	58 (20.7%)	11 (23.9%)	0.62
EHM	97 (34.6%)	12 (26.1%)	0.25
LEN	60 (21.4%)	7 (15.2%)	0.33
ATZ/BV	38 (13.6%)	23 (50.0%)	< 0.01
High total tumor volume	17 (6.1%)	2 (4.4%)	0.64
Adverse event: Hypertension	124 (44.3%)	27 (58.7%)	0.07
Adverse event: Hand-foot syndrome	78 (27.9%)	9 (19.6%)	0.24
Ascites	23 (8.2%)	4 (8.7%)	0.91
History of treatment for HCC	231 (82.5%)	34 (73.9%)	0.17
History of treatment for EV	12 (4.3%)	1 (2.2%)	0.50
PPI	176 (62.9%)	27 (58.7%)	0.59
Findings on contrast enhanced CT			
Diameter of intramural vessel in esophagus ≥ 1.9(mm)	45 (16.1%)	16 (34.8%)	< 0.01
Diameter of portosystemic shunt ≥ 1.8(mm)	94 (33.6%)	22 (47.8%)	0.06
Laboratory data			
Alanine aminotransferases (U/L)	30 (20–50)	35 (25–53)	0.68
Bilirubin (mg/dL)	0.9 (0.7–1.1)	1.0 (0.8–1.2)	0.07
Prothrombin time (international normalized ratio)	1.03 (0.99–1.08)	1.03 (1.01–1.10)	0.92
Albumin (g/dL)	3.8 (3.4–4.1)	3.7 (3.4–4.0)	0.59
Platelets (10^9^/L)	15.3 (11.3–20.3)	15.5 (10.0–19.4)	0.59
Ammonia (μg/dL)	38 (30–55)	35 (29–47)	0.36
Alfa fetoprotein (ng/mL)	52.4 (8.1–932.3)	93.1 (10.6–762.4)	0.34
ALBI score	− 2.48 (− 2.74 to 2.12)	− 2.48 (− 2.74 to 2.12)	0.32
Child-Pugh B	26 (9.3%)	5 (10.8%)	0.73

*ALBI* Albumin-Bilirubin, *ATZ/BEV* atezolizmab/bevacizumab, *CT* computed tomography, *EHM* extrahepatic metastasis, *EV* esophageal varices, *HCC* hepatocellular carcinoma, *LEN* Lenvatinib, *NSAIDs* Non-Steroidal Anti-Inflammatory Drugs, *PD* progression disease, Portosystemic shunt maximum diameter of portosystemic shunt other than esophageal varices, *PPI* Proton pump inhibitor, *PVTT* portal vein tumor thrombosis

**Fig. 2:**
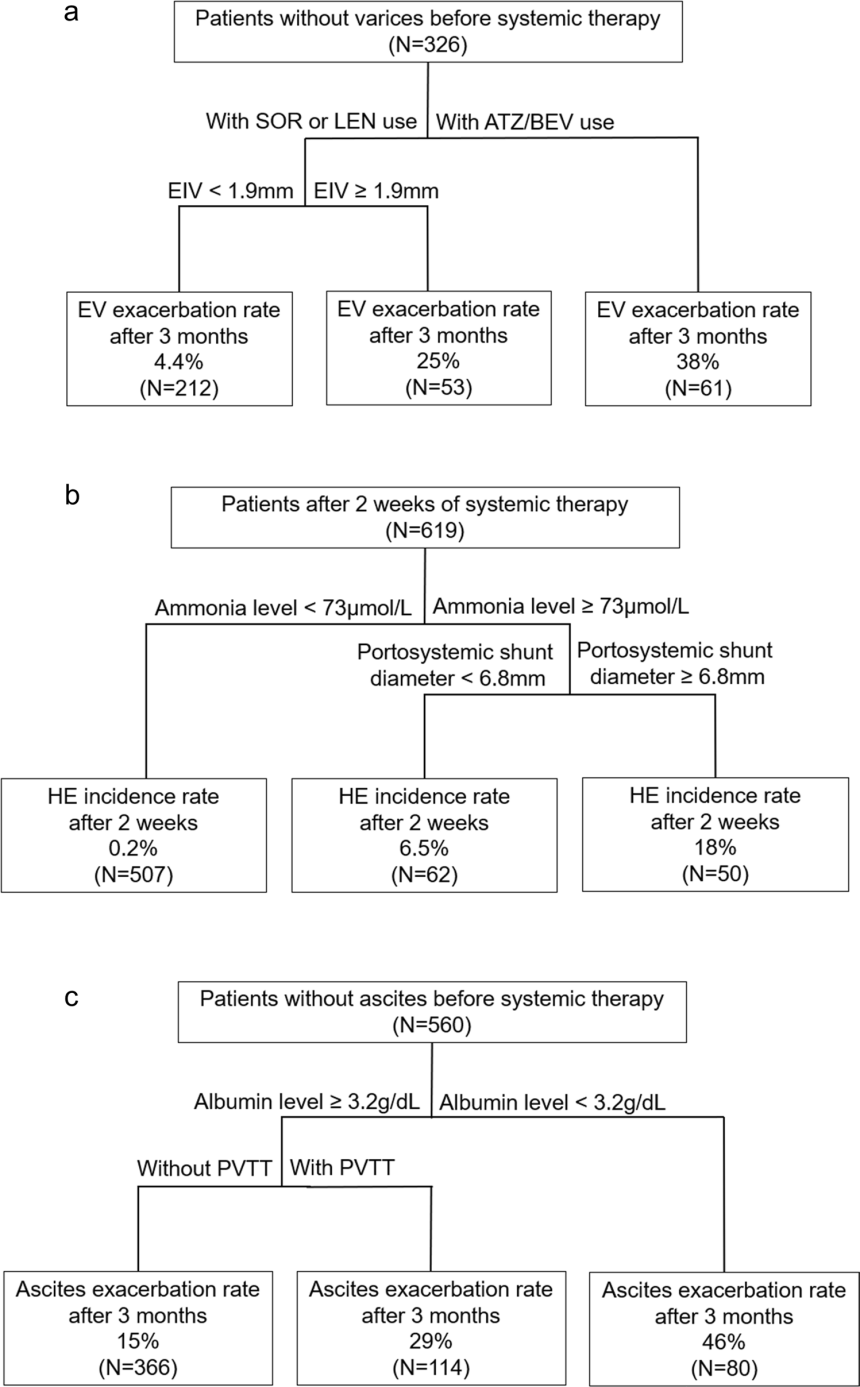
The prognostic model based on the classification and regression tree analysis: **a** EV exacerbation 3 months after systemic therapy. **b** HE incidence within 2 weeks of systemic therapy. **c** Ascites exacerbation 3 months after systemic therapy. *ATZ/BEV* atezolizumab/bevacizumab, *EIV* esophageal intramural vessel, *EV* esophageal varices, *HCC* hepatocellular carcinoma, *HE* hepatic encephalopathy, *LEN* Lenvatinib, *PVTT* portal vein tumor thrombosis, *SOR* sorafenib

To construct a prognostic model, the most relevant variables were selected in the CART analysis and a tree was constructed using an exploratory strategy (Fig. 2a). Patients treated with ATZ/BV were classified as a high-risk group with an EV exacerbation rate of 38.0% (61/326) 3 months after systemic therapy. In the analysis of each type of systemic therapy, EIV diameter was associated with EV exacerbation in the SOR and LEN groups (Supplementary Table 3 and 4). However, there were no significant factors for the exacerbation of EV in Atz/Bev group (Supplementary Table 5).

No association was found between EV exacerbation during systemic therapy and PD after 3 months (*p* = 0.63).

### HE after systemic therapy

Of the 669 patients, ammonia levels were measured before and after 2 weeks of systemic therapy in 619 patients. In these 619 patients, the ammonia levels were significantly elevated after 2 weeks compared to that before systemic therapy (before vs. after 2 weeks; 42.0 [32.0–62.0] μmol/L vs. 50.0 [35.0–80.0] μmol/L, *p* < 0.01). The incidence of HE within 2 weeks of systemic therapy was 2.3%.

The predictors of the incidence of HE within 2 weeks of systemic therapy by univariate analysis are shown in Table 4. Significant predictors of HE incidence were identified in the multivariate analysis, with odds ratios of 1.03 (95% CI 1.01–1.04, *p* < 0.01) for ammonia level and 5.89 (95% CI 1.57–22.10, *p* < 0.01) for portosystemic shunt diameter ≥ 6.8 mm. Ammonia level and portosystemic shunt diameter were also significant factors in analyses restricted to Child–Pugh score 5 patients (Supplementary Table 6).

**Table 4:** Predictors for hepatic encephalopathy within 2 weeks of treatment (univariate analysis)

	Without HE within 2 weeks of treatment	HE within 2 weeks of treatment	*P* value
Number of patients	605	14	
Age (≥75 years)	243 (40.2%)	4 (28.6%)	0.38
Female sex	119 (19.7%)	4 (28.6%)	0.41
Etiology Virus	336 (55.5%)	12 (85.7%)	0.02
Liver cirrhosis	395 (65.3%)	13 (92.9%)	0.03
PVTT	166 (27.4%)	2 (14.3%)	0.27
EHM	187 (30.9%)	4 (28.6%)	0.85
LEN	123 (20.3%)	2 (14.3%)	0.58
ATZ/BEV	85 (14.1%)	0 (0%)	0.13
High total tumor volume	41 (6.8%)	0 (0%)	0.31
Ascites	88 (14.6%)	2 (14.3%)	0.98
History of treatment for HCC	489 (80.8%)	13 (92.9%)	0.26
History of treatment for EV	42 (6.9%)	2 (14.3%)	0.29
PPI	388 (64.1%)	9 (64.3%)	0.99
NSAIDs	81 (13.4%)	0 (0%)	0.44
Findings on contrast enhanced CT			
Diameter of intramural vessel in esophagus ≥ 1.9(mm)	260 (43.0%)	6 (42.9%)	0.99
Diameter of portosystemic shunt ≥ 6.8(mm)	112 (18.5%)	10 (71.4%)	< 0.01
Laboratory data			
Alanine aminotransferases (U/L)	34 (21–55)	32 (25–42)	0.05
Bilirubin (mg/dL)	1.0 (0.7–1.3)	1.3 (0.9–2.0)	0.05
Prothrombin time (international normalized ratio)	1.04 (1.00–1.11)	1.14 (1.10–1.26)	0.01
Albumin (g/dL)	3.6 (0.7–4.0)	3.3 (3.1–3.4)	0.03
Platelets (10^9^/L)	13.6 (9.6–19.4)	10.2 (8.4–10.8)	0.01
Ammonia (μg/dL)	41.0 (32.0–61.0)	89.5 (73.0–121.0)	< 0.01
Alfa fetoprotein (ng/mL)	75.9 (8.3–1884.0)	224.4 (12.3–5117.0)	< 0.01
ALBI score	− 2.28 (− 2.64 to 1.92)	− 1.89 (− 2.08 to 1.68)	0.01
Child-Pugh B	102 (16.9%)	5 (35.7%)	0.07

*ALBI* Albumin-Bilirubin, *ATZ/BEV* atezolizmab/bevacizumab, *CT* computed tomography, *EHM* extrahepatic metastasis, *EV* esophageal varices, *HCC* hepatocellular carcinoma, *LEN* Lenvatinib, *NSAIDs* Non-Steroidal Anti-Inflammatory Drugs, Portosystemic shunt maximum diameter of portosystemic shunt other than esophageal varices, *PPI* Proton pump inhibitor, *PVTT* portal vein tumor thrombosis

The prognostic model for HE occurrence within 2 weeks was constructed in the same manner as that for the prognosis of EV exacerbation (Fig. 2b). According to the decision tree analysis, patients with ammonia levels ≥ 73 μmol/L and portosystemic shunt diameter ≥ 6.8 mm before treatment had an 18% (50/619) higher risk of HE within 2 weeks.

### Ascites exacerbation after 3 months of systemic therapy

Of the 669 patients, 560 patients did not manifest any ascites prior to systemic therapy. Among the 560 patients without ascites, the incidence of ascites 3 months after systemic therapy was approximately 22.3% (125/561).

Table 5 shows the predictors of ascites incidence after 3 months of systemic therapy in patients without ascites via univariate analysis. The multivariate analysis identified significant predictors of ascites incidence, with odds ratios of 2.09 (95% CI 1.31–3.33, *p* < 0.01) for PVTT and 0.47 (95% CI 0.29–0.76, *p* < 0.01) for albumin level. PVTT and albumin level were also significant factors in analyses restricted to Child–Pugh score 5 patients (Supplementary Table 7).

**Table 5:** Predictors for ascites incidence after 3 months of treatment (univariate analysis)

	Without ascites after treatment	With ascites after treatment	*P* value
Number of patients	435	125	
Age (≥ 75 years)	184 (42.3%)	34 (34.4%)	0.11
Female sex	79 (18.2%)	30 (24.0%)	0.15
Liver cirrhosis	265 (60.9%)	95 (76.0%)	< 0.01
PVTT	91 (20.9%)	48 (38.4%)	< 0.01
EHM	125 (28.7%)	44 (35.2%)	0.17
LEN	92 (21.2%)	21 (16.8%)	0.29
ATZ/BEV	63 (14.5%)	13 (10.4%)	0.24
High total tumor volume	13 (3.0%)	11 (8.8%)	< 0.01
Adverse event: hypertension	177 (40.7%)	44 (35.2%)	0.27
Adverse event: hand-foot syndrome	122 (28.1%)	28 (22.4%)	0.21
Etiology Virus	246 (56.6%)	79 (63.2%)	0.18
History of treatment for HCC	375 (86.2%)	98 (78.4%)	0.03
History of treatment for EV	21 (4.8%)	14 (11.2%)	< 0.01
PPI	266 (61.2%)	80 (64.0%)	0.56
Findings on contrast enhanced CT			
Diameter of intramural vessel in esophagus ≥ 1.9(mm)	160 (36.8%)	77 (61.6%) 69 (55.2%)	< 0.01
Diameter of portosystemic shunt ≥ 3.1(mm)	156 (35.9%)	66 (52.8%)	< 0.01
Laboratory data			
Alanine aminotransferases (U/L)	31(20–49)	42 (26–65)	< 0.01
Bilirubin (mg/dL)	0.9 (0.7–1.1)	1.1 (0.8–1.5)	< 0.01
Prothrombin time (international normalized ratio)	1.03 (0.99–1.08)	1.05 (1.00–1.13)	0.08
Albumin (g/dL)	3.8 (3.4–4.1)	3.4 (3.1–3.8)	< 0.01
Platelets (10^9^/L)	13.9 (9.9–19.5)	12.0 (8.4–17.2)	0.50
Ammonia (μg/dL)	39 (31–56)	65 (43–90)	0.10
Alfa fetoprotein (ng/mL)	55.9 (8.5–936.3)	219.7 (30.6–1766.2)	0.06
ALBI score	− 2.44 (− 2.70 to 2.08)	− 2.08 (− 2.39 to 1.74)	<0.01
Child-Pugh B	23 (5.3%)	16 (12.8%)	0.02

*ALBI* Albumin-Bilirubin, *ATZ/BEV* atezolizmab/bevacizumab, *CT* computed tomography, *EHM* extrahepatic metastasis, *EV* esophageal varices, *HCC* hepatocellular carcinoma, *LEN* Lenvatinib, *NSAIDs* Non-Steroidal Anti-Inflammatory Drugs, *PD* progression disease, Portosystemic shunt maximum diameter of portosystemic shunt other than esophageal varices, *PPI* Proton pump inhibitor, *PVTT* portal vein tumor thrombosis

The prognostic model of ascites 3 months after systemic therapy was constructed (Fig. 2c). The incidence rate of ascites after 3 months was 46.0% (80/560) for patients with albumin levels < 3.2 g/dL before treatment and PVTT. In the analysis of each type of systemic therapy, the results of ascites exacerbation in each group were almost similar to the overall results (Supplementary Tables 8–10).

In terms of the effect of systemic therapy for HCC, ascites exacerbation during systemic therapy was associated with PD after 3 months (*p* = 0.02). Classifying PVTT into VP2-3 and VP4, there was no significant difference in ascites incidence between the group with VP2-3 (33.7% [31/92]) and VP4 (36.2% [17/47]).

## Discussion

In patients with HCC undergoing systemic therapy, the management of PH-related complications is vital to effectively prevent treatment interruption secondary to PH-related complications and to maximize treatment benefits. Several studies have evaluated the impact of systemic therapy on PH-related complications [18–21]. However, to the best of our knowledge, only a few studies have identified factors that may contribute to the incidence or exacerbation of PH-related complications during systemic therapy in patients with HCC. In our report, we propose that identifying predictors of PH-related complications prior to treatment may provide an opportunity for prophylactic treatment in patients at a high risk of PH-related complications.

CECT is essential for determining the efficacy of systemic therapy and is frequently performed during treatment to evaluate tumor progression and vascular invasion. Therefore, in terms of invasiveness, and cost efficiency, the evaluation of PH-related complications with CECT would be beneficial. The gold standard for EV screening is endoscopy. However, as our previous and present reports have revealed, CECT has the potential of usefulness in variceal evaluation during systemic therapy for HCC [15]. Furthermore, the portosystemic shunt, a factor in the incidence of HE, can be identified using CECT prior to systemic therapy.

In the literature, some controversy exists regarding the effect of systemic therapy on PH. VEGF produced by hepatocytes and hepatic stellate cells induces angiogenesis in the mesenteric vascular bed and portal circulatory collateral vessels [4], which may exacerbate PH-related complications [5]. The use of anti-VEGF antibodies in rodent models has been reported to reduce portal pressure [6]. Conversely, the inhibition of VEGF affects conserved hepatic sinusoids in non-neoplastic livers, causing sinusoidal changes, and impairment of oxygen and nutrient supply to hepatocytes. This in turn can cause and exacerbate underlying liver disease and PH [8]. Several clinical studies have suggested that SOR may improve PH, while LEN has been reported to worsen PH [7]. Moreover, chronic inflammation may exacerbate PH-related complications as bacterial infection increases portal pressure [8]. In the liver, the innate immune system recognizes damage-associated molecular patterns (DAMPs) and pathogen-associated molecular patterns (PAMPs) via pattern recognition receptors, such as Toll-like receptors, resulting in inflammatory cytokine and reactive oxygen species production [22]. ATZ is an immune checkpoint inhibitor (anti-PD-L1 antibody) that may eliminate DAMPs and PAMPs; however, some studies have reported that the administration of immune checkpoint inhibitors causes liver injury and fibrosis progression [9]. In this study, factors contributing to the incidence and exacerbation of PH-related complications during first-line systemic therapy were explored. In our study, the most accurate and best predictive factor for EV exacerbation after 3 months of treatment was the use of ATZ/BEV. However, ATZ/BEV treatment had no significant effect on the development of HE or the exacerbation of ascites. According to previous reports [23, 24], the HVPG level at which esophageal varices develop is 10.9 mmHg or higher, and the development of ascites has been reported at HVPG levels of 12.5 mmHg or higher, suggesting that the negative effect of ATZ/BEV on PH may first appear in the worsening of esophageal varices. In addition, hepatic encephalopathy is often caused by a low residual intrahepatic venous pressure [25], which was considered less likely to be affected by the negative effect of ATZ/BEV on PH. Thus, our study implies that in patients without EV before systemic therapy, the rapid EV deterioration could be attributed to ATZ/BEV and should be carefully evaluated on CECT during systemic therapy. In our study, the cumulative EV bleeding rate during systemic therapy was 6.4% at 1 year and 11.7% at 2 years. In our previous report, the cumulative EV bleeding rate for patients with early advanced HCC was 3.4% at 1 year and 5.9% at 2 years. A cumulative bleeding rate of 32.4% in patients with EV before systemic therapy was also demonstrated in our study. In another report, the cumulative EV bleeding rate was 12.0% at 2 years in EV patients without HCC [26]. These results suggest that systemic therapy has a negative impact on EV exacerbation and bleeding. Previous reports have also reported that BEV contributes to EV bleeding owing to its effect on PH [27, 28]. Therefore, all patients should undergo endoscopy prior to systemic therapy [28–31]. Patients without PVTT and with an EIV diameter < 3.1 mm could have a relatively low risk of EV bleeding and are less likely to require screening endoscopy before systemic therapy. In this study, no significant difference was found for the EV bleeding rate by type of systemic therapy, although a higher bleeding rate has been reported with the use of ATZ/BEV [28–31]. This may be because more patients in the ATZ/BEV and LEN groups compared to those in the SOR group received prophylactic treatment during systemic therapy (SOR vs. LEN vs. ATZ/BEV 0.5% vs. 3.1% vs. 2.2%. *p* = 0.03).

Limited literature exists regarding the incidence of HE during systemic therapy; however, it has been reported that HE often occurs within 2 weeks upon systemic therapy initiation [32]. In our study, HE incidence within 2 weeks was 2.1%, which is comparable with other published reports (3.8% incidence of HE per 16 days of the median observation period for LEN) [33]. In addition, although PPI administration has been previously reported to be associated with the development of HE [34], this study did not reveal the risk of HE related to the use of PPI. It may be because the development of HE was assessed within 2 weeks, which may have been too short to evaluate the impact of PPI on the development of HE. It has been reported that hyperammonemia is a risk factor for HE [35]. Moreover, ammonia levels peak 2 weeks after systemic therapy [36], and HE is likely to develop a few days after the initiation of systemic therapy [32]. In a previous report from our institution, we reported that hyperammonemia and the presence of a portosystemic shunt were significant factors in the development of HE for LEN [37], which were similarly observed in this study. Therefore, prophylactic medical therapy for HE might be administered early in case of high ammonia levels. This study also demonstrated that a portosystemic shunt of 6.8 mm or greater is a high-risk factor for the development of HE. This concurs with the findings of a previous report that an 8-mm portosystemic shunt diameter is a risk factor for HE [38]. In this study, balloon-occluded retrograde transvenous obliteration (BRTO) might be considered before systemic therapy in high-risk patients. However, validation studies are warranted to determine cases in which BRTO for portosystemic shunts could improve the prognosis of patients during systemic therapy.

One of the most common complications of HCC with cirrhosis is ascites, and the occurrence of ascites is associated with prognosis [39–41]. In our study, the occurrence of ascites at 3 months in patients without ascites prior to systemic therapy was approximately 20.3%, which is considerably higher than that previously reported in patients with cirrhosis (5.1% at 1 year) [42], suggesting that systemic therapy poses a negative influence on the occurrence of ascites. Moreover, PVTT and PD after 3 months of systemic therapy were independent factors for the exacerbation of ascites after 3 months. Cachexia secondary to HCC progression is associated with the development of ascites, similar to other advanced cancers [43].

This study has several limitations. First, a retrospective analysis of data was conducted. Therefore, a prospective evaluation of these data is warranted. Second, this study did not directly establish the correlation between PH-related complications and systemic therapy because of the lack of comparative analysis between groups with and without systemic therapy. Additionally, the evaluation of EV is incomplete based on form factors only. This study did not examine red color signs on endoscopic findings as a bleeding factor for EV, which cannot be evaluated via CECT, and prospective validation is needed in further studies to determine whether red color signs are a significant predictive factor for EV bleeding compared to predictors evaluated by CECT.

In conclusion, systemic therapy may be involved in the exacerbation of PH-related complications during systemic therapy. Therefore, early prophylactic treatment of patients with risk factors for the incidence or exacerbation of PH-related complications should be employed. Additionally, CECT may be useful for the reduction of unnecessary endoscopy and assessment of predictors of PH-related complications.

### Supplementary Information

Below is the link to the electronic supplementary material.

Supplementary file1 (DOC 64 KB)

Supplementary file2 (DOC 64 KB)

Supplementary file3 (DOC 59 KB)

Supplementary file4 (DOC 62 KB)

Supplementary file5 (DOC 62 KB)

Supplementary file6 (DOC 62 KB)

Supplementary file7 (DOC 66 KB)

Supplementary file8 (DOC 60 KB)

Supplementary file9 (DOC 60 KB)

Supplementary file10 (DOC 63 KB)
