# Supplementary material for: Clinical risk factors for portal hypertension-related complications in systemic therapy for hepatocellular carcinoma
Source: J Gastroenterol. 2024 Apr 7;59(6):515–25. doi: 10.1007/s00535-024-02097-9 (PMC11128395; doi:10.1007/s00535-024-02097-9)
Supplement: Supplementary file 1 — Supplementary file1 (DOC 64 KB) [file 535_2024_2097_MOESM1_ESM.doc]

|  | | | | |
| --- | --- | --- | --- | --- |
| Supplementary Table 1. Cox regression analyses of predictive factors for variceal bleeding in the Child-Pugh score 5 group. | | | | |
|  | Univariate hazard ratio  (95% confidence interval) | *P* value | Multivariate hazard ratio  (95% confidence interval) | *P* value |
| Age (≥75 years) | 1.03 (0.35-2.97) | 0.96 | - |  |
| Female sex | 1.25 (0.28-5.59) | 0.77 | - |  |
| Etiology Virus | 2.62 (0.73-9.39) | 0.14 | - |  |
| Etiology Alcohol | 0.69 (0.15-3.08) | 0.63 | - |  |
| Liver cirrhosis | 2.32 (0.73-7.39) | 0.16 | - |  |
| PVTT | 2.67 (0.89-8.09) | 0.08 | - |  |
| EHM | 0.15 (0.02-1.15) | 0.07 | - |  |
| LEN | 0.61 (0.08-4.79) | 0.64 | - |  |
| ATZ/BV | 0 (0) | 0.99 | - |  |
| High total tumor volume | 0 (0) | 0.99 |  |  |
| Ascites | 0 | 1.00 | - |  |
| History of treatment for HCC | 1.33 (0.17-10.27) | 0.79 | - |  |
| History of treatment for EV | 0 (0) | 0.99 | - |  |
| PPI | 0.99 (0.33-2.99) | 0.99 | - |  |
| NSAIDs | 0.77 (0.23-2.65) | 0.68 | - |  |
| Findings on contrast enhanced CT |  |  |  |  |
| Diameter of intramural vessel in esophagus ≥ 3.1(mm) | 30.87 (6.90-138.00) | <0.01 | 17.88 (3.79-84.44) | <0.01 |
| Diameter of portosystemic shunt ≥ 3.0(mm) | 1.58 (0.52-4.75) | 0.42 | - |  |
| Laboratory data |  |  |  |  |
| Alanine aminotransferases (U/L) | 1.01 (0.99-1.02) | 0.57 | - |  |
|  | 3.93 (0.88-17.57) | 0.07 | - |  |
| Prothrombin time (international normalized ratio) | 12.6 (0.32-492.2) | 0.18 | - |  |
| Albumin (g/dL) | 0.60 (0.12-3.11) | 0.54 | - |  |
| Platelets (10^9^/L) | 0.76 (0.64-0.83) | <0.01 | - |  |
| Ammonia (μg/dL) | 1.02 (1.00-1.04) | 0.01 | - |  |
| Alfa fetoprotein (ng/mL) | 1.00 (1.00-1.00) | 0.12 | - |  |
| ALBI score | 3.11 (0.48-20.06) | 0.23 | - |  |
| ALBI; Albumin-Bilirubin, ATZ/BEV; atezolizmab/bevacizumab, CT; computed tomography, EHM; extrahepatic metastasis, EV; esophageal varices, HCC; hepatocellular carcinoma, LEN; Lenvatinib, NSAIDs; Non-Steroidal Anti-Inflammatory Drugs, Portosystemic shunt; maximum diameter of portosystemic shunt other than esophageal varices, PPI; Proton pump inhibitor, PVTT; portal vein tumor thrombosis. | | | | |

| Bilirubin (mg/dL) |
| --- |
