# Supplementary material for: Clinical risk factors for portal hypertension-related complications in systemic therapy for hepatocellular carcinoma
Source: J Gastroenterol. 2024 Apr 7;59(6):515–25. doi: 10.1007/s00535-024-02097-9 (PMC11128395; doi:10.1007/s00535-024-02097-9)
Supplement: Supplementary file 2 — Supplementary file2 (DOC 64 KB) [file 535_2024_2097_MOESM2_ESM.doc]

|  | | | |
| --- | --- | --- | --- |
| Supplementary Table 2. Predictors for EV exacerbation rate after 3 months in the Child-Pugh score 5 group (univariate analysis) | | | |
|  | Without  EV exacerbation  after 3 months | EV exacerbation  after 3 months | *P* value |
| Number of patients | 194 | 28 |  |
| Age (≥75 years) | 79 (40.7%) | 12 (42.9%) | 0.83 |
| Female sex | 28 (14.4%) | 2 (7.1%) | 0.29 |
| Etiology Virus | 114 (58.8%) | 14 (50.0) | 0.38 |
| Etiology Alcohol | 40 (20.6%) | 4 (14.2%) | 0.43 |
| Liver cirrhosis | 90 (46.4%) | 17 (60.7%) | 0.16 |
| PVTT | 44 (22.7%) | 9 (32.1%) | 0.27 |
| EHM | 75 (38.7%) | 7 (25.0%) | 0.16 |
| LEN | 37 (19.1%) | 3 (10.7%) | 0.28 |
| ATZ/BV | 25 (12.9%) | 18 (64.3%) | <0.01 |
| High total tumor volume | 7 (6.1%) | 1 (3.6%) | 0.99 |
| Adverse event: Hypertension | 86 (44.3%) | 18 (66.7%) | 0.03 |
| Adverse event: Hand-foot syndrome | 63 (32.5%) | 3 (10.7%) | 0.02 |
| Ascites | 1 (0.5%) | 0 (0%) | 0.70 |
| History of treatment for HCC | 167 (86.1%) | 21 (75.0%) | 0.13 |
| History of treatment for EV | 5 (2.6%) | 2 (7.1%) | 0.20 |
| PPI | 114 (58.8%) | 15 (53.6%) | 0.60 |
| NSAIDs | 23 (11.9%) | 3 (10.7%) | 0.58 |
| Findings on contrast enhanced CT |  | | |
| Diameter of intramural vessel in esophagus ≥ 1.9(mm) | 28 (14.4%) | 8 (28.6%) | 0.06 |
| Diameter of portosystemic shunt ≥ 1.8(mm) | 55 (28.4%) | 14 (50.0%) | 0.02 |
| Laboratory data |  | | |
| Alanine aminotransferases (U/L) | 30 (20-50) | 35 (25-53) | 0.70 |
| | Bilirubin (mg/dL) | | --- | | 0.9 (0.7-1.1) | 1.0 (0.8-1.2) | 0.24 |
| Prothrombin time (international normalized ratio) | 1.03 (0.99-1.08) | 1.03 (1.01-1.10) | 0.77 |
| Albumin (g/dL) | 3.8 (3.4-4.1) | 3.7 (3.4-4.0) | 0.95 |
| Platelets (10^9^/L) | 15.3 (11.3-20.3) | 15.5 (10.0-19.4) | 0.98 |
| Ammonia (μg/dL) | 38 (30-55) | 35 (29-47) | 0.52 |
| Alfa fetoprotein (ng/mL) | 52.4 (8.1-932.3) | 93.1 (10.6-762.4) | 0.51 |
| ALBI score | -2.48 (-2.74--2.12) | -2.48 (-2.74--2.12) | 0.70 |
| ALBI; Albumin-Bilirubin, ATZ/BEV; atezolizmab/bevacizumab, CT; computed tomography, EHM; extrahepatic metastasis, EV; esophageal varices, HCC; hepatocellular carcinoma, LEN; Lenvatinib, NSAIDs; Non-Steroidal Anti-Inflammatory Drugs, PD; progression disease, Portosystemic shunt; maximum diameter of portosystemic shunt other than esophageal varices, PPI; Proton pump inhibitor, PVTT; portal vein tumor thrombosis. | | | |
