# Supplementary material for: Clinical risk factors for portal hypertension-related complications in systemic therapy for hepatocellular carcinoma
Source: J Gastroenterol. 2024 Apr 7;59(6):515–25. doi: 10.1007/s00535-024-02097-9 (PMC11128395; doi:10.1007/s00535-024-02097-9)
Supplement: Supplementary file 3 — Supplementary file3 (DOC 59 KB) [file 535_2024_2097_MOESM3_ESM.doc]

|  | | | |
| --- | --- | --- | --- |
| Supplementary Table 3. Predictors for EV exacerbation rate after 3 months in the SOR group (univariate analysis) | | | |
|  | Without  EV exacerbation  after 3 months | EV exacerbation  after 3 months | *P* value |
| Number of patients | 182 | 16 |  |
| Age (≥75 years) | 69 (37.9%) | 9 (56.3%) | 0.15 |
| Female sex | 33 (18.1%) | 1 (6.3%) | 0.23 |
| Etiology Virus | 104 (57.1%) | 13 (81.3%) | 0.06 |
| Liver cirrhosis | 92 (50.6%) | 11 (68.8%) | 0.16 |
| PVTT | 32 (17.6%) | 5 (31.3%) | 0.18 |
| EHM | 71 (39.0%) | 5 (31.3%) | 0.54 |
| High total tumor volume | 5 (2.8%) | 1 (6.3%) | 0.57 |
| Adverse event: Hypertension | 54 (29.7%) | 6 (37.5%) | 0.51 |
| Adverse event: Hand-foot syndrome | 65 (35.7%) | 8 (50.0%) | 0.26 |
| Ascites | 14 (7.7%) | 2 (12.5%) | 0.50 |
| History of treatment for HCC | 164 (90.1%) | 14 (87.5%) | 0.74 |
| History of treatment for EV | 7 (3.9%) | 1 (6.3%) | 0.64 |
| PPI | 131 (72.0%) | 13 (81.3%) | 0.42 |
| Findings on contrast enhanced CT |  |  |  |
| Diameter of intramural vessel in esophagus ≥ 1.9(mm) | 32 (17.6%) | 10 (62.5%) | <0.01 |
| Diameter of portosystemic shunt ≥ 1.8(mm) | 62 (34.1%) | 4 (25.0%) | 0.46 |
| Laboratory data |  |  |  |
| Alanine aminotransferases (U/L) | 39 (25-61) | 44 (31-68) | 0.54 |
| | Bilirubin (mg/dL) | | --- | | 0.9 (0.6-1.1) | 1.1 (0.9-1.4) | 0.12 |
| Prothrombin time (international normalized ratio) | 1.03 (0.99-1.09) | 1.10 (1.05-1.15) | 0.45 |
| Albumin (g/dL) | 3.9 (3.5-4.2) | 3.6 (3.4-4.2) | 0.26 |
| Platelets (10^9^/L) | 14.5 (10.4-20.3) | 10.0 (7.5-16.3) | 0.26 |
| Ammonia (μg/dL) | 38 (31-55) | 35 (26-49) | 0.57 |
| Alfa fetoprotein (ng/mL) | 71.2 (12.3-1003.0) | 418.0 (13.2-1876.9) | 0.56 |
| ALBI score | -2.53 (-2.81--2.22) | -2.22 (-2.68--2.05) | 0.13 |
| Child-Pugh B | 17 (9.3%) | 2 (12.5%) | 0.68 |
| ALBI; Albumin-Bilirubin, CT; computed tomography, EHM; extrahepatic metastasis, EV; esophageal varices, HCC; hepatocellular carcinoma, NSAIDs; Non-Steroidal Anti-Inflammatory Drugs, PD; progression disease, Portosystemic shunt; maximum diameter of portosystemic shunt other than esophageal varices, PPI; Proton pump inhibitor, PVTT; portal vein tumor thrombosis, SOR; sorafenib. | | | |
|  | | | |
