# Supplementary material for: Clinical risk factors for portal hypertension-related complications in systemic therapy for hepatocellular carcinoma
Source: J Gastroenterol. 2024 Apr 7;59(6):515–25. doi: 10.1007/s00535-024-02097-9 (PMC11128395; doi:10.1007/s00535-024-02097-9)
Supplement: Supplementary file 4 — Supplementary file4 (DOC 62 KB) [file 535_2024_2097_MOESM4_ESM.doc]

|  | | | |
| --- | --- | --- | --- |
| Supplementary Table 4. Predictors for EV exacerbation rate after 3 months in the LEN group (univariate analysis) | | | |
|  | Without  EV exacerbation  after 3 months | EV exacerbation  after 3 months | *P* value |
| Number of patients | 60 | 7 |  |
| Age (≥75 years) | 26 (21.7%) | 4 (57.1%) | 0.43 |
| Female sex | 13 (21.7%) | 0 (0%) | 0.17 |
| Etiology Virus | 34 (56.7%) | 4 (57.1%) | 0.98 |
| Liver cirrhosis | 38 (63.3%) | 7 (100.0%) | 0.05 |
| PVTT | 20 (33.3%) | 3 (42.9%) | 0.62 |
| EHM | 16 (26.7%) | 3 (42.9%) | 0.37 |
| High total tumor volume | 4 (6.7%) | 0 (0%) | 0.48 |
| Adverse event: Hypertension | 39 (65.0%) | 4 (57.1%) | 0.68 |
| Adverse event: Hand-foot syndrome | 13 (21.7%) | 1 (14.3%) | 0.65 |
| Ascites | 4 (6.7%) | 1 (14.3%) | 0.47 |
| History of treatment for HCC | 46 (76.7%) | 5 (71.4%) | 0.76 |
| History of treatment for EV | 4 (6.7%) | 0 (0%) | 0.48 |
| PPI | 33 (55.0%) | 3 (42.9%) | 0.54 |
| Findings on contrast enhanced CT |  | | |
| Diameter of intramural vessel in esophagus ≥ 1.9(mm) | 8 (13.3%) | 3 (42.9%) | 0.046 |
| Diameter of portosystemic shunt ≥ 1.8(mm) | 20 (33.3%) | 5 (71.4%) | 0.049 |
| Laboratory data |  | | |
| Alanine aminotransferases (U/L) | 27 (16-46) | 47 (20-60) | 0.67 |
| | Bilirubin (mg/dL) | | --- | | 1.0 (0.8-1.2) | 1.2 (0.9-1.6) | 0.29 |
| Prothrombin time (international normalized ratio) | 1.03 (0.99-1.08) | 1.11 (1.03-1.35) | 0.02 |
| Albumin (g/dL) | 3.5 (3.3-4.10) | 3.6 (3.0-4.0) | 0.36 |
| Platelets (10^9^/L) | 15.8 (12.1-22.3) | 16.2 (13.1-19.1) | 0.64 |
| Ammonia (μg/dL) | 43 (34-60) | 34 (29-40) | 0.33 |
| Alfa fetoprotein (ng/mL) | 47.0 (7.5-1068.6) | 239.2 (4.8-16097.0) | 0.89 |
| ALBI score | -2.25 (-2.65--1.97) | -2.11 (-2.62—1.66) | 0.21 |
| Child-Pugh B | 5 (8.3%) | 2 (28.6%) | 0.10 |
| ALBI; Albumin-Bilirubin, CT; computed tomography, EHM; extrahepatic metastasis, EV; esophageal varices, HCC; hepatocellular carcinoma, LEN; Lenvatinib, NSAIDs; Non-Steroidal Anti-Inflammatory Drugs, PD; progression disease, Portosystemic shunt; maximum diameter of portosystemic shunt other than esophageal varices, PPI; Proton pump inhibitor, PVTT; portal vein tumor thrombosis. | | | |
