# Supplementary material for: Clinical risk factors for portal hypertension-related complications in systemic therapy for hepatocellular carcinoma
Source: J Gastroenterol. 2024 Apr 7;59(6):515–25. doi: 10.1007/s00535-024-02097-9 (PMC11128395; doi:10.1007/s00535-024-02097-9)
Supplement: Supplementary file 5 — Supplementary file5 (DOC 62 KB) [file 535_2024_2097_MOESM5_ESM.doc]

|  | | | |
| --- | --- | --- | --- |
| Supplementary Table 5. Predictors for EV exacerbation rate after 3 months in the ATZ/BEV group (univariate analysis) | | | |
|  | Without  EV exacerbation  after 3 months | EV exacerbation  after 3 months | *P* value |
| Number of patients | 38 | 23 |  |
| Age (≥75 years) | 23 (60.5%) | 12 (52.2%) | 0.52 |
| Female sex | 7 (18.4%) | 3 (13.0%) | 0.58 |
| Etiology Virus | 12 (31.6%) | 7 (30.4%) | 0.93 |
| Liver cirrhosis | 20 (52.6%) | 14 (60.9%) | 0.53 |
| PVTT | 6 (15.8%) | 3 (13.0%) | 0.77 |
| EHM | 10 (26.3%) | 4 (17.4%) | 0.42 |
| High total tumor volume | 8 (21.1%) | 1 (4.4%) | 0.07 |
| Adverse event: Hypertension | 31 (81.6%) | 17 (73.9%) | 0.48 |
| Adverse event: Hand-foot syndrome | 0 (0%) | 0 (0%) | - |
| Ascites | 5 (13.2%) | 1 (4.4%) | 0.26 |
| History of treatment for HCC | 21 (55.3%) | 15 (65.2%) | 0.44 |
| History of treatment for EV | 0 (0%) | 0 (0%) | - |
| PPI | 12 (31.6%) | 11 (47.8%) | 0.20 |
| Findings on contrast enhanced CT |  | | |
| Diameter of intramural vessel in esophagus ≥ 1.9(mm) | 5 (13.2%) | 3 (13.0%) | 0.99 |
| Diameter of portosystemic shunt ≥ 1.8(mm) | 12 (31.6%) | 13 (56.5%) | 0.05 |
| Laboratory data |  | | |
| Alanine aminotransferases (U/L) | 27 (19-43) | 30 (19-44) | 0.70 |
| | Bilirubin (mg/dL) | | --- | | 0.9 (0.7-1.2) | 0.9 (0.8-1.0) | 0.83 |
| Prothrombin time (international normalized ratio) | 1.00 (0.97-1.04) | 1.01 (0.97-1.03) | 0.71 |
| Albumin (g/dL) | 3.8 (3.5-4.0) | 3.8 (3.6-4.0) | 0.46 |
| Platelets (10^9^/L) | 16.6 (12.5-21.8) | 17.0 (13.2-21.7) | 0.74 |
| Ammonia (μg/dL) | 32 (25-44) | 35 (30-47) | 0.17 |
| Alfa fetoprotein (ng/mL) | 10.2 (3.7-107.6) | 42.7 (8.0-246.6) | 0.93 |
| ALBI score | -2.39 (-2.70--2.11) | -2.52 (-2.69--2.25) | 0.94 |
| Child-Pugh B | 4 (10.5%) | 1 (4.4%) | 0.40 |
| ALBI; Albumin-Bilirubin, ATZ/BEV; atezolizmab/bevacizumab, CT; computed tomography, EHM; extrahepatic metastasis, EV; esophageal varices, HCC; hepatocellular carcinoma, NSAIDs; Non-Steroidal Anti-Inflammatory Drugs, PD; progression disease, Portosystemic shunt; maximum diameter of portosystemic shunt other than esophageal varices, PPI; Proton pump inhibitor, PVTT; portal vein tumor thrombosis. | | | |
