# Supplementary material for: Clinical risk factors for portal hypertension-related complications in systemic therapy for hepatocellular carcinoma
Source: J Gastroenterol. 2024 Apr 7;59(6):515–25. doi: 10.1007/s00535-024-02097-9 (PMC11128395; doi:10.1007/s00535-024-02097-9)
Supplement: Supplementary file 6 — Supplementary file6 (DOC 62 KB) [file 535_2024_2097_MOESM6_ESM.doc]

|  | | | |
| --- | --- | --- | --- |
| Supplementary Table 6. Predictors for hepatic encephalopathy within 2 weeks of treatment in the Child-Pugh score 5 group (univariate analysis) | | | |
|  | Without HE within 2 weeks of treatment | HE within 2  weeks of treatment | *P* value |
| Number of patients | 309 | 4 |  |
| Age (≥75 years) | 125 (40.5%) | 1 (25.0%) | 0.38 |
| Female sex | 51 (16.5%) | 1 (25.0%) | 0.65 |
| Etiology Virus | 184 (59.6%) | 3 (75.0%) | 0.53 |
| Etiology Alcohol | 56 (18.1%) | 0 (0%) | 0.35 |
| Liver cirrhosis | 168 (54.4%) | 4 (100%) | 0.07 |
| PVTT | 78 (25.2%) | 1 (25.0%) | 0.99 |
| EHM | 105 (34.0%) | 2 (50.0%) | 0.50 |
| LEN | 54 (17.5%) | 0 (0%) | 0.36 |
| ATZ/BEV | 47 (15.2%) | 0 (0%) | 0.40 |
| High total tumor volume | 12 (3.9%) | 0 (0%) | 0.69 |
| Ascites | 1 (0.3%) | 0 (0%) | 0.91 |
| History of treatment for HCC | 264 (85.4%) | 4 (100.0%) | 0.41 |
| History of treatment for EV | 10 (3.2%) | 1 (25.0%) | 0.02 |
| PPI | 189 (61.2%) | 2 (50.0%) | 0.65 |
| NSAIDs | 34 (11.0%) | 0 (0%) | 0.19 |
| Findings on contrast enhanced CT |  | | |
| Diameter of intramural vessel in esophagus ≥ 1.3(mm) | 137 (44.3%) | 3 (75.0%) | 0.22 |
| Diameter of portosystemic shunt ≥ 6.8(mm) | 42 (13.6%) | 2 (50.0%) | 0.04 |
| Laboratory data |  | | |
| Alanine aminotransferases (U/L) | 33 (21-52) | 26 (23-33) | 0.50 |
| | Bilirubin (mg/dL) | | --- | | 0.9 (0.7-1.2) | 0.9 (0.9-1.3) | 0.40 |
| Prothrombin time (international normalized ratio) | 1.03 (0.99-1.08) | 1.14 (1.09-1.18) | <0.01 |
| Albumin (g/dL) | 3.7 (3.4-4.1) | 3.4 (3.2-3.6) | 0.20 |
| Platelets (10^9^/L) | 14.1 (10.0-19.9) | 10.2 (8.8-10.7) | 0.34 |
| Ammonia (μg/dL) | 39.0 (31.0-56.0) | 105.0 (77.0-122.0) | <0.01 |
| Alfa fetoprotein (ng/mL) | 85.5 (11.0-1515.9) | 177.0 (6.3-5117.0) | 0.03 |
| ALBI score | -2.39 (-2.68—2.09) | -2.05 (-2.28--1.89) | 0.18 |
| ALBI; Albumin-Bilirubin, ATZ/BEV; atezolizmab/bevacizumab, CT; computed tomography, EHM; extrahepatic metastasis, EV; esophageal varices, HCC; hepatocellular carcinoma, LEN; Lenvatinib, NSAIDs; Non-Steroidal Anti-Inflammatory Drugs, Portosystemic shunt; maximum diameter of portosystemic shunt other than esophageal varices, PPI; Proton pump inhibitor, PVTT; portal vein tumor thrombosis. | | | |
