# Supplementary material for: Clinical risk factors for portal hypertension-related complications in systemic therapy for hepatocellular carcinoma
Source: J Gastroenterol. 2024 Apr 7;59(6):515–25. doi: 10.1007/s00535-024-02097-9 (PMC11128395; doi:10.1007/s00535-024-02097-9)
Supplement: Supplementary file 7 — Supplementary file7 (DOC 66 KB) [file 535_2024_2097_MOESM7_ESM.doc]

|  | | | |
| --- | --- | --- | --- |
| Supplementary Table 7. Predictors for ascites incidence after 3 months of treatment in the Child-Pugh score 5 group (univariate analysis) | | | |
|  | Without ascites after treatment | With ascites  after treatment | *P* value |
| Number of patients | 283 | 52 |  |
| Age (≥ 75 years) | 120 (42.4%) | 17 (32.7%) | 0.19 |
| Female sex | 42 (14.8%) | 11 (21.2%) | 0.25 |
| Liver cirrhosis | 149 (52.7%) | 36 (69.2%) | 0.03 |
| PVTT | 59 (20.9%) | 21 (40.4%) | <0.01 |
| EHM | 96 (33.9%) | 21 (40.4%) | 0.37 |
| LEN | 52 (18.4%) | 4 (7.7%) | 0.06 |
| ATZ/BEV | 46 (16.2%) | 5 (9.6%) | 0.22 |
| High total tumor volume | 9 (3.2%) | 3 (5.8%) | 0.36 |
| Adverse event: Hypertension | 126 (44.5%) | 17 (32.7%) | 0.11 |
| Adverse event: Hand-foot syndrome | 86 (30.4%) | 15 (28.9%) | 0.82 |
| Etiology Virus | 163 (57.6%) | 36 (69.2%) | 0.12 |
| Etiology Alcohol | 54 (19.1%) | 6 (11.5%) | 0.19 |
| History of treatment for HCC | 247 (87.3%) | 41 (78.9%) | 0.11 |
| History of treatment for EV | 5 (1.2%) | 7 (13.5%) | <0.01 |
| PPI | 164 (58.0%) | 31 (59.6%) | 0.82 |
| NSAIDs | 30 (10.6%) | 8 (15.4%) | 0.03 |
| Findings on contrast enhanced CT |  | | |
| Diameter of intramural vessel in esophagus ≥ 1.7(mm) | 97 (34.3%) | 26 (50.0%) | 0.03 |
| Diameter of portosystemic shunt ≥ 3.1(mm) | 79 (27.9%) | 20 (38.5%) | 0.13 |
| Laboratory data |  | | |
| Alanine aminotransferases (U/L) | 31(20-49) | 37 (24-68) | 0.04 |
|  | 0.9 (0.7-1.1) | 1.1 (0.9-1.4) | <0.01 |
| Prothrombin time (international normalized ratio) | 1.03 (0.99-1.08) | 1.04 (1.01-1.14) | 0.03 |
| Albumin (g/dL) | 3.8 (3.4-4.1) | 3.5 (3.2-3.9) | 0.04 |
| Platelets (10^9^/L) | 14.2 (10.0-20.1) | 13.3 (9.6-18.2) | 0.98 |
| Ammonia (μg/dL) | 39 (30-55) | 42 (33-66) | 0.41 |
| Alfa fetoprotein (ng/mL) | 55.5 (8.5-1068.6) | 220.6 (24.2-4238.0) | 0.06 |
| ALBI score | -2.48 (-2.71--2.14) | -2.11 (-2.47--1.81) | <0.01 |
| ALBI; Albumin-Bilirubin, ATZ/BEV; atezolizmab/bevacizumab, CT; computed tomography, EHM; extrahepatic metastasis, EV; esophageal varices, HCC; hepatocellular carcinoma, LEN; Lenvatinib, NSAIDs; Non-Steroidal Anti-Inflammatory Drugs, PD; progression disease, Portosystemic shunt; maximum diameter of portosystemic shunt other than esophageal varices, PPI; Proton pump inhibitor, PVTT; portal vein tumor thrombosis. | | | |

| Bilirubin (mg/dL) |
| --- |
