# Supplementary material for: Clinical risk factors for portal hypertension-related complications in systemic therapy for hepatocellular carcinoma
Source: J Gastroenterol. 2024 Apr 7;59(6):515–25. doi: 10.1007/s00535-024-02097-9 (PMC11128395; doi:10.1007/s00535-024-02097-9)
Supplement: Supplementary file 8 — Supplementary file8 (DOC 60 KB) [file 535_2024_2097_MOESM8_ESM.doc]

|  | | | |
| --- | --- | --- | --- |
| Supplementary Table 8. Predictors for ascites incidence after 3 months of treatment in the SOR group (univariate analysis) | | | |
|  | Without ascites after treatment | With ascites  after treatment | *P* value |
| Number of patients | 280 | 91 |  |
| Age (≥ 75 years) | 116 (41.4%) | 26 (28.6%) | 0.03 |
| Female sex | 52 (18.6%) | 23 (25.3%) | 0.17 |
| Liver cirrhosis | 168 (60.0%) | 67 (73.6%) | 0.02 |
| PVTT | 57 (20.4%) | 29 (31.9%) | 0.02 |
| EHM | 87 (31.1%) | 36 (39.6%) | 0.14 |
| High total tumor volume | 5 (1.8%) | 8 (8.8%) | <0.01 |
| Adverse event: Hypertension | 86 (30.7%) | 23 (25.3%) | 0.32 |
| Adverse event: Hand-foot syndrome | 102 (36.4%) | 27 (29.7%) | 0.24 |
| Etiology Virus | 170 (60.7%) | 64 (70.3%) | 0.10 |
| History of treatment for HCC | 264 (94.0%) | 80 (87.9%) | 0.06 |
| History of treatment for EV | 13 (4.6%) | 8 (8.8%) | 0.13 |
| PPI | 197 (70.4%) | 63 (69.2%) | 0.84 |
| Findings on contrast enhanced CT |  | | |
| Diameter of intramural vessel in esophagus ≥ 1.9(mm) | 113 (40.4%) | 52 (57.1%) | 0.01 |
| Diameter of portosystemic shunt ≥ 3.1(mm) | 98 (35.0%) | 45 (49.5%) | 0.01 |
| Laboratory data |  | | |
| Alanine aminotransferases (U/L) | 33 (19-54) | 37 (25-58) | 0.07 |
|  | 0.9 (0.6-1.2) | 1.1 (0.8-1.5) | <0.01 |
| Prothrombin time (international normalized ratio) | 1.04 (1.00-1.10) | 1.01 (1.00-1.16) | 0.41 |
| Albumin (g/dL) | 3.7 (3.4-4.1) | 3.5 (3.2-3.9) | <0.01 |
| Platelets (10^9^/L) | 12.8 (9.2-19.6) | 13.1 (8.4-17.2) | 0.62 |
| Ammonia (μg/dL) | 39 (31-60) | 42 (33-64) | 0.34 |
| Alfa fetoprotein (ng/mL) | 99.8 (11.4-1430.9) | 294.9 (32.7-4584.0) | 0.18 |
| ALBI score | -2.45 (-2.69--2.08) | -2.11 (-2.47--1.77) | <0.01 |
| Child-Pugh B | 18 (6.4%) | 10 (11.0%) | 0.15 |
| ALBI; Albumin-Bilirubin, CT; computed tomography, EHM; extrahepatic metastasis, EV; esophageal varices, HCC; hepatocellular carcinoma, NSAIDs; Non-Steroidal Anti-Inflammatory Drugs, PD; progression disease, Portosystemic shunt; maximum diameter of portosystemic shunt other than esophageal varices, PPI; Proton pump inhibitor, PVTT; portal vein tumor thrombosis, SOR; sorafenib. | | | |

| Bilirubin (mg/dL) |
| --- |
