# Supplementary material for: Clinical risk factors for portal hypertension-related complications in systemic therapy for hepatocellular carcinoma
Source: J Gastroenterol. 2024 Apr 7;59(6):515–25. doi: 10.1007/s00535-024-02097-9 (PMC11128395; doi:10.1007/s00535-024-02097-9)
Supplement: Supplementary file 9 — Supplementary file9 (DOC 60 KB) [file 535_2024_2097_MOESM9_ESM.doc]

|  | | | |
| --- | --- | --- | --- |
| SupplementaryTable 9. Predictors for ascites incidence after 3 months of treatment in the LEN group (univariate analysis) | | | |
|  | Without ascites after treatment | With ascites  after treatment | *P* value |
| Number of patients | 92 | 21 |  |
| Age (≥ 75 years) | 33 (35.9%) | 9 (42.9%) | 0.55 |
| Female sex | 18 (19.6%) | 5 (23.8%) | 0.66 |
| Liver cirrhosis | 60 (65.2%) | 18 (85.7%) | 0.07 |
| PVTT | 25 (21.2%) | 11 (52.4%) | 0.03 |
| EHM | 22 (23.9%) | 5 (23.8%) | 0.99 |
| High total tumor volume | 2 (2.2%) | 1 (4.8%) | 0.51 |
| Adverse event: Hypertension | 42 (45.7%) | 10 (47.6%) | 0.87 |
| Adverse event: Hand-foot syndrome | 20 (21.7%) | 1 (4.8%) | 0.07 |
| Etiology Virus | 53 (57.6%) | 9 (42.9%) | 0.22 |
| History of treatment for HCC | 73 (79.4%) | 11 (52.4%) | 0.01 |
| History of treatment for EV | 5 (5.4%) | 4 (19.1%) | 0.04 |
| PPI | 45 (48.9%) | 11 (52.4%) | 0.77 |
| Findings on contrast enhanced CT |  | | |
| Diameter of intramural vessel in esophagus ≥ 1.9(mm) | 31 (33.7%) | 9 (42.9%) | 0.43 |
| Diameter of portosystemic shunt ≥ 3.1(mm) | 35 (38.0%) | 14 (66.7%) | 0.02 |
| Laboratory data |  | | |
| Alanine aminotransferases (U/L) | 28 (19-43) | 32 (22-59) | 0.17 |
| | Bilirubin (mg/dL) | | --- | | 0.9 (0.7-1.3) | 1.2 (1.0-1.4) | 0.03 |
| Prothrombin time (international normalized ratio) | 1.03 (0.99-1.08) | 1.05 (1.01-1.13) | 0.33 |
| Albumin (g/dL) | 3.7 (3.3-4.1) | 3.2 (2.9-3.4) | <0.01 |
| Platelets (10^9^/L) | 14.8 (11.0-19.9) | 13.5 (11.4-18.4) | 0.60 |
| Ammonia (μg/dL) | 43 (32-60) | 49 (35-82) | 0.19 |
| Alfa fetoprotein (ng/mL) | 33.6 (7.4-473.3) | 1094.4 (64.0-4457.8) | 0.36 |
| ALBI score | -2.28 (-2.68--1.99) | -1.81 (-2.09--1.63) | <0.01 |
| Child-Pugh B | 4 (4.4%) | 4 (19.1%) | 0.02 |
| ALBI; Albumin-Bilirubin, CT; computed tomography, EHM; extrahepatic metastasis, EV; esophageal varices, HCC; hepatocellular carcinoma, LEN; Lenvatinib, NSAIDs; Non-Steroidal Anti-Inflammatory Drugs, PD; progression disease, Portosystemic shunt; maximum diameter of portosystemic shunt other than esophageal varices, PPI; Proton pump inhibitor, PVTT; portal vein tumor thrombosis. | | | |
