# Supplementary material for: Clinical risk factors for portal hypertension-related complications in systemic therapy for hepatocellular carcinoma
Source: J Gastroenterol. 2024 Apr 7;59(6):515–25. doi: 10.1007/s00535-024-02097-9 (PMC11128395; doi:10.1007/s00535-024-02097-9)
Supplement: Supplementary file 10 — Supplementary file10 (DOC 63 KB) [file 535_2024_2097_MOESM10_ESM.doc]

|  | | | |
| --- | --- | --- | --- |
| Supplementary Table 10. Predictors for ascites incidence after 3 months of treatment in the ATZ/BEV group (univariate analysis) | | | |
|  | Without ascites after treatment | With ascites  after treatment | *P* value |
| Number of patients | 63 | 13 |  |
| Age (≥ 75 years) | 35 (55.7%) | 8 (61.5%) | 0.69 |
| Female sex | 9 (14.3%) | 2 (15.4%) | 0.92 |
| Liver cirrhosis | 37 (58.7%) | 10 (76.9%) | 0.22 |
| PVTT | 9 (14.3%) | 8 (61.5%) | <0.01 |
| EHM | 16 (25.4%) | 3 (23.1%) | 0.86 |
| High total tumor volume | 6 (9.5%) | 2 (15.4%) | 0.53 |
| Adverse event: Hypertension | 49 (77.8%) | 11 (84.6%) | 0.58 |
| Adverse event: Hand-foot syndrome | 0 (0%) | 0 (0%) | - |
| Etiology Virus | 23 (36.5%) | 6 (46.2%) | 0.51 |
| History of treatment for HCC | 39 (61.9%) | 7 (53.9%) | 0.59 |
| History of treatment for EV | 3 (4.8%) | 2 (15.4%) | 0.16 |
| PPI | 24 (38.1%) | 6 (46.2%) | 0.59 |
| Findings on contrast enhanced CT |  | | |
| Diameter of intramural vessel in esophagus ≥ 1.9(mm) | 16 (25.4%) | 8 (61.5%) | 0.01 |
| Diameter of portosystemic shunt ≥ 3.1(mm) | 23 (36.5%) | 7 (53.9%) | 0.24 |
| Laboratory data |  | | |
| Alanine aminotransferases (U/L) | 31(22-41) | 34 (26-94) | 0.10 |
|  | 0.9 (0.7-1.1) | 1.0 (0.8-1.5) | 0.16 |
| Prothrombin time (international normalized ratio) | 1.01 (0.97-1.04) | 1.06 (1.00-1.09) | <0.01 |
| Albumin (g/dL) | 3.9 (3.5-4.1) | 3.3 (3.0-3.9) | 0.03 |
| Platelets (10^9^/L) | 16.5 (11.8-21.7) | 17.1 (13.4-20.9) | 0.63 |
| Ammonia (μg/dL) | 33 (27-53) | 37 (34-59) | 0.44 |
| Alfa fetoprotein (ng/mL) | 18.4 (4.2-186.8) | 15.7 (8.1-685.7) | 0.22 |
| ALBI score | -2.48 (-2.73--2.24) | -1.92 (-2.50--1.71) | <0.01 |
| Child-Pugh B | 1 (2.0%) | 2 (15.4%) | 0.02 |
| ALBI; Albumin-Bilirubin, ATZ/BEV; atezolizmab/bevacizumab, CT; computed tomography, EHM; extrahepatic metastasis, EV; esophageal varices, HCC; hepatocellular carcinoma, NSAIDs; Non-Steroidal Anti-Inflammatory Drugs, PD; progression disease, Portosystemic shunt; maximum diameter of portosystemic shunt other than esophageal varices, PPI; Proton pump inhibitor, PVTT; portal vein tumor thrombosis. | | | |

| Bilirubin (mg/dL) |
| --- |
